# Broadband Polarization Conversion Metasurface Based on Metal Cut-Wire Structure for Radar Cross Section Reduction

**DOI:** 10.3390/ma11040626

**Published:** 2018-04-19

**Authors:** Jia Ji Yang, Yong Zhi Cheng, Chen Chen Ge, Rong Zhou Gong

**Affiliations:** 1 School of Optical and Electronic Information, Huazhong University of Science and Technology, Wuhan 430074, China; yangjiajialnow@163.com (J.J.Y.); gec@hust.edu.cn (C.C.G.); 2 School of Information Science and Engineering, Wuhan University of Science and Technology, Wuhan 430081, China

**Keywords:** coding metasurface, polarization conversion, energy scattering, RCS reduction

## Abstract

A class of linear polarization conversion coding metasurfaces (MSs) based on a metal cut-wire structure is proposed, which can be applied to the reduction properties of radar cross section (RCS). We firstly present a hypothesis based on the principle of planar array theory, and then verify the RCS reduction characteristics using linear polarization conversion coding MSs by simulations and experiments. The simulated results show that in the frequency range of 6–14 GHz, the linear polarization conversion ratio reaches a maximum value of 90%, which is in good agreement with the theoretical predictions. For normal incident *x*- and *y*-polarized waves, RCS reduction of designed coding MSs 01/01 and 01/10 is essentially more than 10 dB in the above-mentioned frequency range. We prepare and measure the 01/10 coding MS sample, and find that the experimental results in terms of reflectance and RCS reduction are in good agreement with the simulated ones under normal incidence. In addition, under oblique incidence, RCS reduction is suppressed as the angle of incidence increases, but still exhibits RCS reduction effects in a certain frequency range. The designed MS is expected to have valuable potential in applications for stealth field technology.

## 1. Introduction

As a subwavelength artificial composite, metamaterial (MM) has some exotic electromagnetic (EM) properties that are unavailable in nature [1,2,3,4]. Research on MM has inspired many novel applications, including perfect lens imaging, inverse doppler effect, abnormal tunneling and many other phenomena [5,6,7,8,9,10]. As a two-dimensional (2D) planar form of MM [11,12,13,14,15], metasurfaces (MS) are constituted of subwavelength element arrays and have superior performance in tailoring the EM waves, which can be used to control the reflection/refraction wavefront in a smaller size range. By designing an artificial structure on the interface, the propagation and polarization characteristics of the EM wave regulation can be realized [16,17,18,19,20,21]. Therefore, the MS has prospects for potential application in polarization manipulation [22,23,24,25,26,27,28,29], high-performance antennas [30,31], and so on [32,33,34,35,36,37,38,39].

Radar stealth technology is concerned with reducing the target radar echo signal to achieve stealth, and radar cross section (RCS) area is an important physical quantity for measuring the radar echo capability of a target. RCS can be effectively reduced by using a MS designed with a suitable supper-unit structure [40,41,42]. As an important branch of MS, phase gradient MS (GMS) can be used to reduce the RCS by introducing an artificial wave vector at an in-plane direction to control the propagation direction of transmitted and reflected wave beams. Wu et al. proposed a GMS based on a cruciform structure, which could be applied to RCS reduction at low frequency ranges [43]. This designed MS has the defect of a narrow band range.

Recently, the concept of “coding MS” based on GMS was proposed by Cui et al. [44], and indicates a new manner for designing MS. The basic units are arranged on a matrix, and the phase responses of elements like 0 and π can be regarded as digitally “0” and “1”. Coding MS can diffuse the energy of EM waves in each direction by optimizing the matrix arrangement. This coding MS exhibits a broadband characteristic compared with traditional GMS, but still has the disadvantage of polarization-sensitivity. Based on the above research, we designed a series of coding MSs, one of which has the characteristics of a wide band range and polarization insensitivity, meaning that it promotes the RCS reduction of incident EM waves in all polarization directions. The traditional absorber can absorb electromagnetic waves and convert them into heat energy, which will be detected by infrared devices. The RCS reduction mechanism of the designed MS is different from the absorber; by designing the structure graphics and coding scheme, the incident waves are reflected irregularly back into free space, which lowers the probability of the MS being detected by infrared devices. Therefore, the scattering energy of each directional beam is small, which can achieve an effective RCS reduction at different angles [45,46,47,48,49,50,51,52].

The metal cut-wire structure (rectangular strip structure) is advantageous in terms of its simple structure, the adjustability of its geometric parameters, and has a wide application with respect to the design of MMs and MSs. In this work, we apply it as the basic unit structure for the design of a broadband linear polarization conversion MS. We can obtain the required phase difference by rotating the metal cut-wire structure by 90° along the propagation direction, and the phase response of the two basic units are 0 and π, denoted by “0” and “1”, respectively. At the same time, we design a class of 1-bit coding form to focus on the RCS reduction characteristics of the polarization conversion MS. The designed MS presents the characteristics of a broadband range, polarization-insensitivity, and wide incidence angle. For normal *x*/*y*-polarized incidence, both simulations and experiments show that the RCS of the MS is reduced by an average of 10 dB in the frequency range of 6–14 GHz. Meanwhile, at oblique incidence, RCS reduction is suppressed, but still has a certain effect in the above-mentioned frequency range.

## 2. Design of Basic Unit and Theoretical Analysis

**Figure 1:**
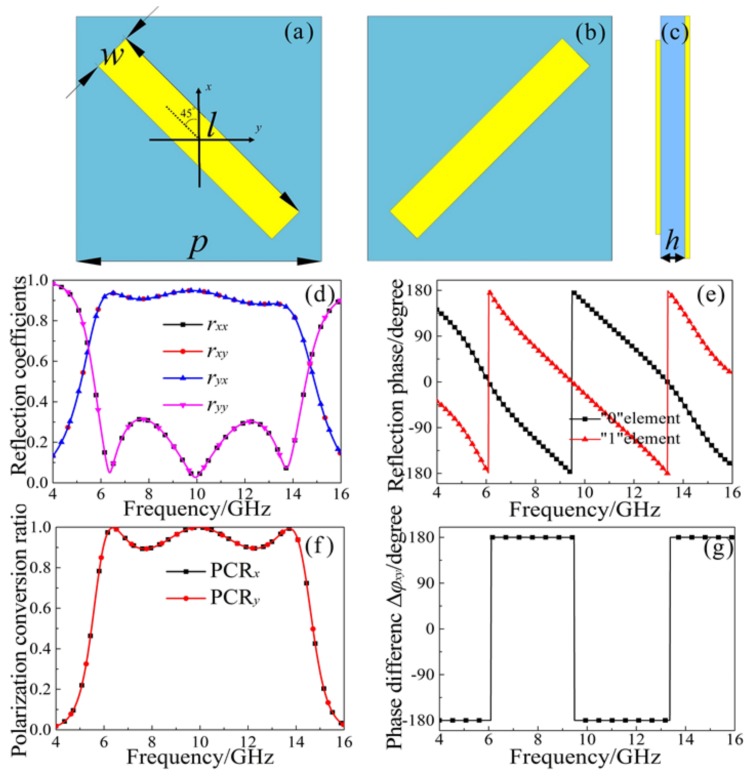
(**a**) The element “0” with an angle of 45° to the *x*-axis; (**b**) the element “1” rotated 90° counterclockwise about the *z*-axis; (**c**) the side view of the basic unit; (**d**) the reflection coefficient of elements “0” and “1” under normal *x*- and *y*-polarized incidence; (**e**) the reflection phase of cross-polarized wave; (**f**) the linear polarization conversion ratio of *x*- and *y*-polarized wave; (**g**) cross-polarization reflection phase difference of elements “0” and “1”.

In this paper, we present a basic unit based on a metal cut-wire structure, which we assume to be a “0” unit, as shown in Figure 1a. The whole unit-cell is divided into three functional layers, where the period is *p* = 10 mm. This design of the three functional layers can form a Fabry-Perot-like resonance cavity, consequently leading to interference of cross-polarization couplings in multi-reflection [21,22]. The front layer is the metal cut-wire structure of copper film; the length and width are *l* = 10 mm and *w* = 1.6 mm, respectively. The front layer cut-wire structure possesses a symmetric axis at 45° with respect to the *x* or *y* direction, such that a 90° polarization conversion can be achieved when the incident wave is *x*- or *y*-polarized. The middle layer dielectric substrate is FR4 film with a thickness of *h* = 3.5 mm; the side view is shown in Figure 1c. The dielectric constant is 4.3, and the loss tangent is 0.025. The back layer is continuous copper film and has the same thickness as the front metal structure, which is 0.035 mm. We rotated the metal cut-wire counterclockwise by 90° (Figure 1a) to obtain the MS unit structure, as shown in Figure 1b, which is set to “1”.

We used the frequency domain solver on the EM simulation software CST MICROWAVE STUDIO to obtain the co-polarization (*r_xx_* and *r_yy_*) and cross-polarization (*r_yx_* and *r_xy_*) reflection coefficients for both incident *x*-polarized and *y*-polarized waves. As suggested by the reflection coefficients in Figure 1d, the structure is able to achieve efficient linear polarization conversion across a wide frequency range of 6–14 GHz, the cross-polarization reflection coefficients (*r_yx_* and *r_xy_*) are greater than 0.85, and the co-polarization reflection coefficients (*r_xx_* and *r_yy_*) are substantially less than 0.3. In addition, at resonance frequencies, the co-polarization reflection coefficients (*r_xx_* and *r_yy_*) reach a minimum, and the corresponding amplitudes are less than 0.1. The corresponding cross-polarization reflection coefficients (*r_yx_* and *r_xy_*) reach a maximum, and the amplitudes are greater than 0.9. This result indicates that the normal incident *x*(*y*)-polarized waves are almost completely converted to *y* (*x*)-polarized waves, or produced approximately 90° linear polarization deflections.

In order to visually reflect the polarization conversion capability of the MS, we define the polarization conversion efficiency as follows [22,23]: PCR*_x_* = |*r_yx_*|^2^/(|*r_yx_*|^2^ + |*r_xx_*|^2^) and PCR*_y_* = |*r_xy_*|^2^/(|*r_xy_*|^2^ + |*r_yy_*|^2^). In Figure 1f, the linear polarization conversion ratio of the *x*- and *y*-polarized waves are as high as 90% and reached 99% at resonance frequencies. Figure 1e,g shows the cross-polarization phases of the “0” and “1” units, and the corresponding phase difference, Δ*φ*_10_(Δ*φ_xy_*), in the whole 4–16 GHz range. It is observed that the cross-polarization phase difference Δ*φ*_10_(Δ*φ_xy_*) = ±180° can be obtained, which indicates that the phase gradient of the designed MS is 180°.

**Figure 2:**
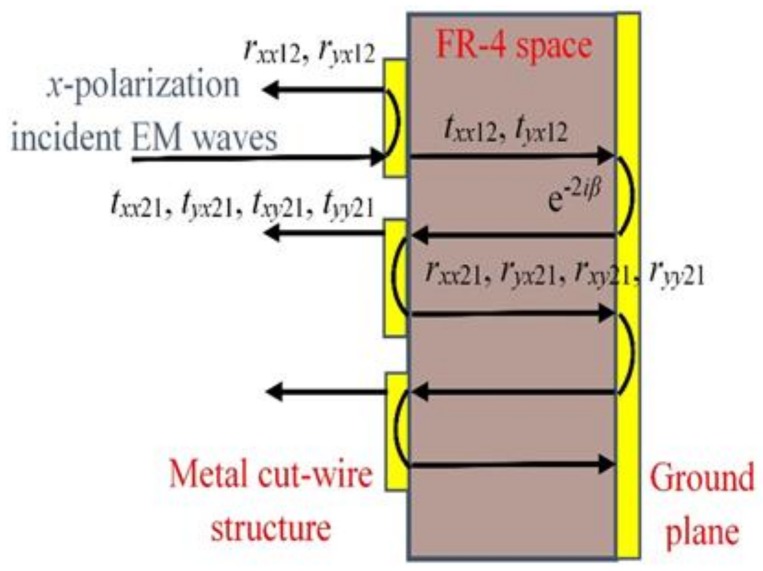
Schematic of multiple reflections and transmissions in a Fabry-Perot-like resonance cavity for polarization conversion.

**Figure 3:**
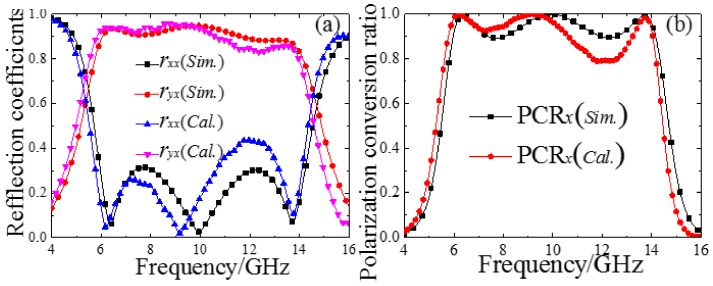
Simulated, calculated (**a**) reflection coefficients and (**b**) PCRs of the designed MS.

This basic unit has excellent simulated results; mainly due to the front cut-wire structure layer and metal back layer forming a Fabry-Perot-like resonance cavity, we are able to observe the multiple reflections and transmissions in the Fabry-Perot-like resonance cavity as shown in Figure 2. The transmitted EM waves continue to travel in the dielectric spacer until they encounter the ground plane with a complex propagation phase $\beta =\sqrt{\varepsilon_{r}}k_{0}d$, where *k*_0_ is the free space wavenumber, the $\varepsilon_{r}$ and *d* are the relative permittivity and thickness of the middle dielectric layer. The partial reflection and transmission waves arrive at the air-spacer interface again from the reverse direction after the additional propagation phase *β* in the dielectric spacer [22]. The incident EM wave prompted multiple reflections in this resonance cavity, including co-polarization wave interference cancellation, and cross-polarization wave interference superposition. Based on our previous research [22], the cross-polarization and co-polarization reflection coefficients can be expressed as: $r_{yx}={\vec{r}}_{yx}+\sum_{j=1}^{\infty }r_{yj}$ and $r_{xx}={\vec{r}}_{xx}+\sum_{j=1}^{\infty }r_{xj}$. Thus, we simulate the unit-cell structure without the ground plane, and use MATLAB software to calculate the reflection coefficients and polarization conversion ratio for the *x*-polarized wave incidence. As shown in Figure 3a,b, the calculated data are in reasonable agreement with the simulated data, further illustrating the working principle of the Fabry-Perot-like resonance cavity. Therefore, we can use the characteristics of the ±180° cross-polarization reflected phase difference based on the metal cut-wire structure elements “0” and “1” and realize polarization conversion by combining different coding combinations.

**Figure 4:**
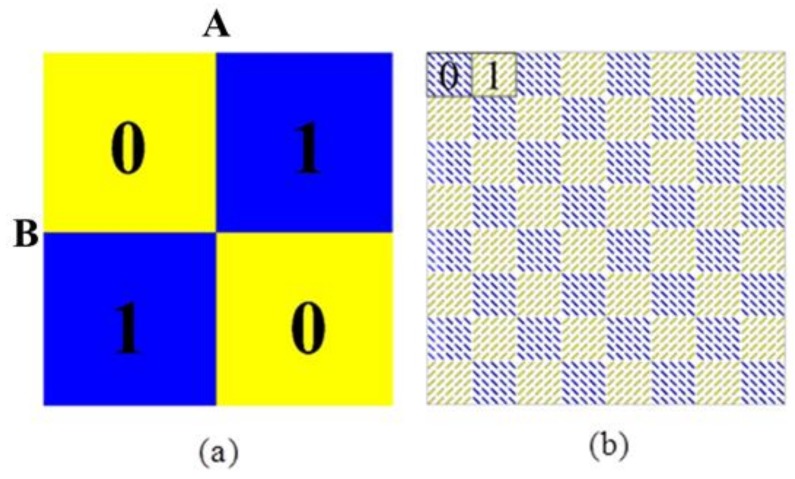
(**a**) 2 × 2 structure arrangement of the 01/10 coding MS; (**b**) illustration of the 01/10 coding MS; the “0” and “1” indicate the super-cells, and are distinguished by blue and yellow colors; each super-cell consists of 5 × 5 unit-cells of “0” and “1”, shown in Figure 1a,b.

A traditional MS has a uniform reflection phase when a plane wave impinges on it, leading to a strong scattering pattern. In order to manipulate the reflected beam direction, a phase gradient is introduced into the interface to control the equiphase wavefront. To suppress the normal strong scattering of the MS, the simplest way is to generate a matrix of designed phase distribution. We propose a series of polarization conversion coding MSs composed of “0” and “1” units, and make a hypothesis based on the principle of planar array theory [53,54]; then, we verify this derivation using data from the simulation. For a MS with A × B elements, the total scattering field can be expressed as [53]:

(1) $$E_{total}=EF\cdot AF$$

where *EF* represents the scattering field of the cell pattern; and *AF* represents the array factor, which can be expressed as [53]:

(2) $$AF=\sum_{a=1}^{A}\sum_{b=1}^{B}e^{j[(a-1/2)(kd\;\sin\theta \cos\varphi )+(b-1/2)(kd\;\sin\theta \sin\varphi )+\phi (a,b)]}$$

where *θ* is the angle between the incident wave and the *z*-axis along the *XOZ*-plane; *φ* is the angle between incident wave and *x*-axis along the *XOY*-plane; *a* and *b* represent the rows and columns of unit cell; *k* = 2*π*/*λ*, *λ* is the wavelength of incident wave; *d* is the distance between the units; and *Φ* (*a*, *b*) is the phase difference between the elements. As shown in Figure 4a, we put 01/10 code into 2 × 2 structure arrangement, each super-cell consists of 5 × 5 basic units; the scattering field can be expressed as:

(3) $$E_{total}=EF_{0}\cdot AF_{0}+EF_{1}\cdot AF_{1}$$

where *AF*_0_ and *AF*_1_ are the elements “0” and “1”, respectively; and *EF*_0_ and *EF*_1_ are the scattering fields of the “0” and “1” elements, respectively. The array factor *AF*_2×2_ with a 2 × 2 arrangement can be expressed as:

(4) $$\begin{array}{l} AF_{2\times 2}=[e^{j[\frac{1}{2}kd\sin\theta \cos\varphi +\frac{1}{2}kd\sin\theta \sin\varphi +\phi (1,1)]}+e^{j[\frac{3}{2}kd\sin\theta \cos\varphi +\frac{3}{2}kd\sin\theta \sin\varphi +\phi (2,2)}]\\ +[e^{j[\frac{1}{2}kd\sin\theta \cos\varphi +\frac{3}{2}kd\sin\theta \sin\varphi +\phi (1,2)]}+e^{j[\frac{3}{2}kd\sin\theta \cos\varphi +\frac{1}{2}kd\sin\theta \sin\varphi +\phi (2,1)}] \end{array}$$

For the 01/10 coding sequence, *Φ*(1,1) and *Φ*(2,2) correspond to the phase difference of 0°; *Φ*(1,2) is the 180° phase difference of two adjacent cells of column B, and *Φ*(1,2)→2*kdsinθsinφ*; *Φ*(2,1) is the 180° phase difference of two adjacent cells of row A, and *Φ*(1,2)→2*kdsinθcosφ*. Further derivation shows that the array factor of coding 01/10 is:

(5) AF01/10=[ejkd(sinθcosφ+sinθsinφ)2+e−jkd(sinθcosφ+sinθsinφ)2]+[ejkd(sinθsinφ−sinθcosφ)2+e−jkd(sinθsinφ−sinθcosφ)2]

**Figure 5:**
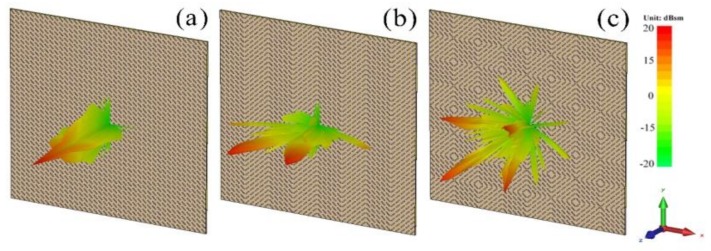
Simulation far-field patterns of the scattering of (**a**) 00/00 or 11/11; (**b**) 01/01 or 10/10; and (**c**) 01/10 at 10 GHz for the coding MS.

The above formula can be decomposed according to the arrangement of Figure 5a:

(6) {AF0=ejkd(sinθcosφ+sinθsinφ)2+e−jkd(sinθcosφ+sinθsinφ)2AF1=ejkd(sinθsinφ−sinθcosφ)2+e−jkd(sinθsinφ−sinθcosφ)2

*AF*_0_ and *AF*_1_ are expressed in the matrices as shown below:

(7) [AF0AF1]=[ejkdsinθcosφ2e−jkdsinθcosφ2e−jkdsinθcosφ2ejkdsinθcosφ2]×[ejkdsinθsinφ2e−jkdsinθsinφ2]

We can see that the first half of the matrix corresponds to the arrangement of the 01/10 coding structure, and the latter part of the matrix corresponds to the 0–1 phase difference distribution.

For the 01/01 coding sequence, the derivation method is similar to the one for the 01/10 coding sequence. For 2 × 2 coding MS, the area of the unit-cell structure is assumed to be 1. For normal incidence, the angles of incidence are *θ* = *φ* = 0°, coding 01/10 corresponds to *AF*_0_ = *AF*_1_ = 2/4 = 1/2. Similarly, coding 01/01 corresponds to *AF*_0_ = *AF*_1_ = 1/2.

Since the unit-cell structure has the characteristic of polarization conversion, the polarization direction of the incident wave is along the *x*-axis. For the normal incident field *EF_t_*, the incident fields *EF*_0_ and *EF*_1_ are expressed as:

(8) $$\left\{ \begin{array}{l} EF_{0}=r_{x_{0}x_{0}}EF_{t}+r_{y_{0}x_{0}}EF_{t}\\ EF_{1}=r_{x_{1}x_{1}}EF_{t}+r_{y_{1}x_{1}}EF_{t} \end{array}\right.$$

where *r_xx_* = $\vec{r_{xx}}e^{\vec{\varphi_{xx}}}$ is the co-polarization reflection coefficient and *r_yx_* = $\vec{r_{yx}}e^{\vec{\varphi_{yx}}}$ is the cross-polarization reflection coefficient. From the previous simulated results, we can see that the units “0” and “1” have the same co-polarization reflection phase, and the cross-polarization reflection phase difference is 180°. Therefore, the total scattering field can be expressed as:

(9) $$\begin{array}{l} E_{total}=\frac{1}{2}(\vec{r_{x_{0}x_{0}}}EF_{t}e^{\vec{\varphi_{x_{0}x_{0}}}}+\vec{r_{x_{1}x_{1}}}EF_{t}e^{\vec{\varphi_{x_{1}x_{1}}}}+\vec{r_{y_{0}x_{0}}}EF_{t}e^{\vec{\varphi_{y_{0}x_{0}}}}+\vec{r_{y_{1}x_{1}}}EF_{t}e^{\vec{\varphi_{y_{1}x_{1}}}})\\ =\frac{1}{2}(\vec{r_{x_{0}x_{0}}}EF_{t}e^{\vec{\varphi_{x_{0}x_{0}}}}+\vec{r_{x_{1}x_{1}}}EF_{t}e^{\vec{\varphi_{x_{1}x_{1}}}})=\vec{r_{xx}}EF_{t}e^{\vec{\varphi_{xx}}} \end{array}$$

The size of the scattering field of the array structure is |*E_total_*| = *r_xx_*|*EF_t_*|. For a metal plate, the size of the scattering field is |*EF^i^_total_*| = |*EF_t_*|, and the scattering coefficient is *r_xx_* = |*E_total_*|**/**|*EF^i^_total_*|. We assume that the RCS reduction of the MS is greater than 10 dB, so the following condition needs to be met:

(10) $$-10\mathrm{lg}{\left|r_{xx}\right|}^{2}\geq 10\;\mathrm{dB}\Rightarrow r_{xx}\leq \sqrt{0.1}\approx 0.316$$

In summary, coding 01/01 and 01/10 have better RCS reduction characteristics under certain conditions compared with coding 00/00 and 11/11. Since the co-polarization and cross-polarization phases of 00/00 and 00/11 are consistent, the cross-polarization components of the scattering field can’t be offset, and RCS can’t be reduced; thus, the corresponding RCS reduction is 0. The next step is to verify this hypothesis by simulation.

To meet the periodic boundary conditions required for simulation, we used a 5 × 5 basic unit as a super-cell and designed a series of coding forms to explore the polarization conversion characteristics of the MS. Figure 5a–c shows the far-field scattering characteristic diagram of each regular coding form at 10 GHz. The energy scattering direction of coding 00/00 or 11/11 is upright, and the normal scattering capability is very strong, suggesting that these coding MSs do not have characteristics of RCS reduction under normal incidence. The energy scattering of 01/01 and 10/10 are the same, diverging to both sides. The energy scattering of 01/10 diverges all around, and the normal scattering capacity is relatively weak. Thus, it can be expected that the 01/01 and 01/10 coding MSs have normal RCS reduction characteristics. Therefore, by controlling the coding method of the MS, it is possible to change the scattering direction of the energy. However, the coding 01/10 has polarization-insensitive properties compared to the coding 01/01. In order to meet the needs of practical applications, we focus on studying the MS of coding 01/10, and the schematic diagram is shown in Figure 4b.

## 3. Simulation, Experiment and Discussion

**Figure 6:**
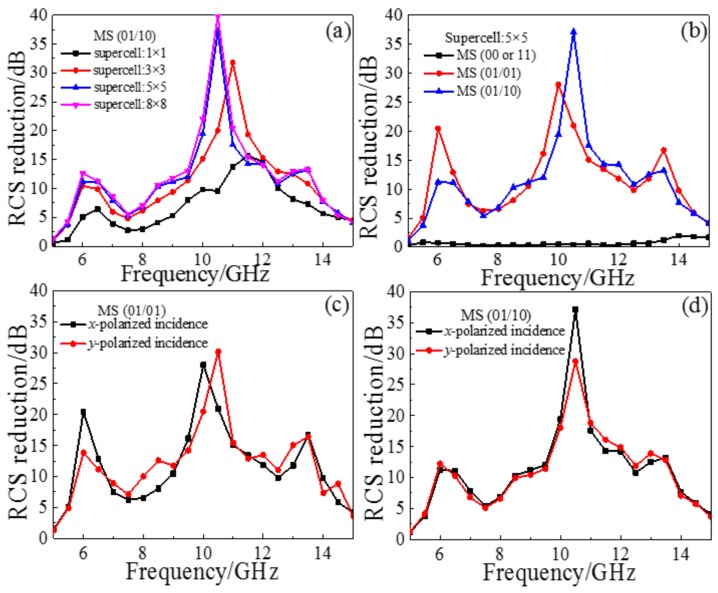
Simulated results of a series of regular coding MSs: (**a**) RCS reduction of 01/10 coding MS for different super-cell combinations; (**b**) RCS reduction of the different regular coding MS with 5 × 5 super-cell; (**c**) RCS reduction of the 01/01 coding MS for normal *x*- and *y*-polarized incident waves; (**d**) RCS reduction of the 01/10 coding MS for normal incident *x*- and *y*-polarized waves.

Using the frequency domain solver in the EM simulation software CST MICROWAVE STUDIO (2016, CST, Darmstadt, Germany), we perform a numerical simulation of three regular coding MSs, as shown in Figure 4. Firstly, for 01/10 coding MS, the RCS reduction characteristics of a super-cell with n × n basic units were discussed numerically; as shown in Figure 6a, the RCS reduction of 5 × 5 super-cell increases compared with a 3 × 3 or 1 × 1 super-cell, and the overall curves shift to the lower frequencies. With an increase in the number of units, the numerical RCS reduction gradually stabilizes. To meet the requirements of test conditions and practical application, a 5 × 5 supercell was selected as the basic coding unit for studying the RCS reduction characteristics. Figure 6b shows the RCS reduction in all arrangements under normal incidence, where the MSs of coding 01/01 and 01/10 achieve RCS reduction in a broadband range. In Figure 1d, the reflection coefficient of the basic unit is *r_xx_* = 0.318 > 0.316 at the 7.7 GHz peak, and *r_xx_* = 0.301 < 0.316 at the 12.2 GHz peak. According to Formula (10), we can see that the RCS reduction of the MS should be less than 10 dB around 7.7 GHz. Figure 6b also shows that the MSs of coding 01/01 and 01/10 have dips at the above-mentioned frequency, where RCS reduction is less than 10 dB around 7.7 GHz. For the MSs of coding 00/00 and 11/11, it can be seen that the RCS reduction is essentially zero in our frequency range of interest of 5–15 GHz. This is mainly due to the consistency of co-polarization and cross-polarization phases for all basic units, where the RCS of the target can’t be reduced since the cross-polarization components of the scattering field can’t be offset. These simulated results near perfectly verify the above analysis.

We simulated the RCS reduction of 01/01 and 01/10 coding MSs for the incidence of *x*-polarized and *y*-polarized waves, as shown in Figure 6c,d, respectively. In Figure 6c, the RCS reduction amplitude of the 01/01 coding MS is clearly different between the *x*- and *y*-polarized waves under normal incidence, indicating that the 01/01 coding MS has a certain polarized selection characteristic. As shown in Figure 6d, the RCS reduction curves of the 01/10 coding MS under *x*- and *y*-polarized waves are slightly different, but the trend is basically consistent under normal incidence, indicating that the coding MS exhibits a polarization-insensitive property. The 01/10 coding MS exhibits a near polarization-insensitive property, mainly due to its chessboard arrangement; the direction of electric field and magnetic field are relatively consistent with the incident *x*- and *y*-polarized waves. Hence, we can see the reflection coefficients of EM waves are basically the same. However, the 01/01 is asymmetric, and the direction of electric field and magnetic field are not consistent with the incident *x*- and *y*-polarized waves under the same incident surface. In addition, the RCS reduction of the 01/10 coding MS is greater than 10 dB on average throughout a wide frequency range of 6–14 GHz and is greater than 20 dB in the frequency range of 10–11 GHz. At 10.5 GHz, the RCS reduction reacches a maximum of 37 dB.

**Figure 7:**
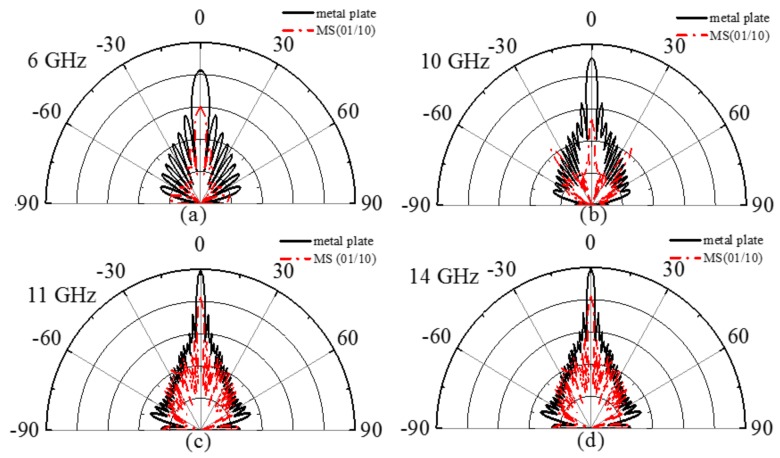
Scattering patterns of the 01/10 coding MS and metal plate in the *XOZ*-plane at (**a**) 6 GHz; (**b**) 10 GHz; (**c**) 11 GHz; and (**d**) 14 GHz.

To further study the RCS reduction characteristics of 01/10 coding MS, we study the scattering pattern of the *XOZ*-plane under normal incidence, as shown in Figure 7a–d; where the scattering characteristics of the 01/10 coding MSs at 6 GHz, 10 GHz, 11 GHz, and 14 GHz are compared with a metal plate with the same size of 400 × 400 mm^2^. According to the law of energy conservation, the main lobe energy is suppressed significantly by enhancing the scattered energy of the side lobes to achieve RCS reduction under normal incidence. The pattern shows that the metal plate has a main lobe throughout the whole band under normal incidence. As shown in Figure 7a,d, relative to the metal plate, the MS has a certain inhibitory effect on the main lobe at 6 and 14 GHz, respectively. As shown in Figure 7b,c, the MS has an obvious inhibitory effect on the main lobe at 10 and 11 GHz, respectively. Based on the above results, it can be suggested that the polarization conversion MS can realize RCS reduction throughout a wide frequency range and can adjust the scattering field dynamically.

**Figure 8:**
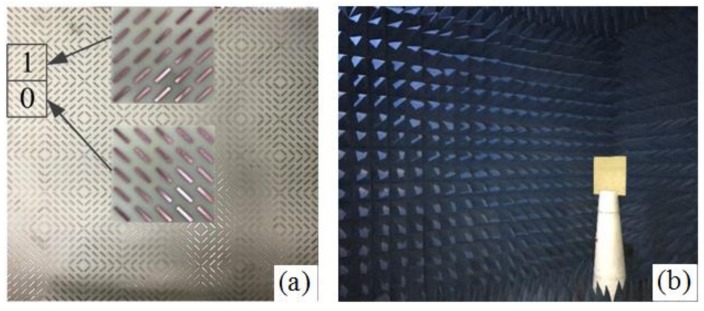
(**a**) The fabricated 01/10 coding MS sample; (**b**) the measurement setup in the microwave anechoic chamber.

In order to further verify the RCS reduction characteristics of the polarization conversion coding MS, we fabricated a MS sample of 01/10 coding and measured it in a microwave anechoic chamber. A 400 × 400 mm^2^ sample was fabricated using traditional printed circuit board (PCB) technology, as shown in Figure 8a. The front and back layers of the sample are covered with copper, and the middle layer is a FR4 substrate with a thickness of 3.5 mm. Using the free space method, we measured the sample in the microwave anechoic chamber (see Figure 8b). The measured sample was fixed in the center of a rotating foam tower, the transmitter and receiver antennas were fixed to the same height, ensuring an angle of 5°. Then, we connected two horn antennas of co-polarization state to two ports of the Agilent Technologies N5244A Vector Analyzer and measured the RCS of the sample.

**Figure 9:**
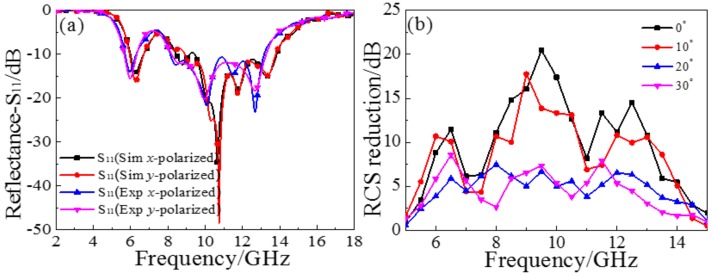
(**a**) The simulated and measured reflectance results of the sample; (**b**) RCS reduction of the sample under oblique incident waves from 0° to 30°.

The reflectance of simulation and experiment under normal incident *x*- and *y*-polarized wave are shown in Figure 9a. Under the conditions of incident different polarization waves, the simulated results under *x*- and *y*-polarized incidence are basically coincident, as are the measured results, which reveals the near polarization insensitivity of the proposed MS. However, the results of the simulation and experiments are quite different at the main peak. The main reason behind this deviation may be the error occurring in the production of the sample, such as the inability to precisely control the thickness, or the sample not being completely flat. The simulated and measured reflectance (*x*- and *y*-polarization) near 7.7 GHz is slightly greater than −10 dB, which verifies the previous theoretical analysis, and the reflectance (*x*- and *y*-polarization) is below −5 dB in the frequency range of 5–14 GHz. Figure 9b shows the RCS reduction of the measured sample at different angles of incidence. At normal incidence, although there is a valley less than 10 dB near the 7.7 GHz, the RCS reduction is basically greater than 10 dB in the frequency range of 6–14 GHz. Under a small oblique incident angle, the RCS reduction curves of oblique incident angles from 0° to 10° are basically the same. When the incident angles reach 20° and 30°, RCS reduction fluctuate around 5 dB in the frequency range of 6–14 GHz. It can be seen that RCS reduction is suppressed as the angle increases, but still has a certain effect within the above frequency range.

## 4. Conclusions

In this work, a basic unit based on a metal cut-wire structure was designed, which allowed us to achieve the highly efficient conversion of linear polarization within a broadband frequency range. A hypothesis was proposed based on the principle of planar array theory and was verified by using the simulated data of the MS. Utilizing a super-cell consisting of 5 × 5 basic units, we studied the RCS reduction of the polarization conversion MS based on super-coding theory. By simply rotating the metal cut-wire structure, it is possible to realize the characteristics of “0” and “1” basic unit 180° cross-polarization reflected phase differences. This method avoids the need to change the graphic design size and makes it possible to arrange super-coding in an easy and efficient way. We simulated a series of coding forms to generate the RCS reduction graph and scattering pattern under normal incidence, further exploring the relationship of the coding MS with different arrangement modes and the energy scattering direction at the same time. The influence of polarization conversion characteristics for RCS reduction was also proved. Based on the simulated design, we fabricated a 01/10 coding MS sample and measured its reflectance and RCS reduction. The measured results are in good agreement with the simulation, which proves that the polarization conversion MS structure is able to realize the reduction of RCS. Our work proposes a new design as a basis for future studies on RCS reduction of MSs, which has potential application in stealth field technology.
